# Systematic Characterization of Expression Profiles and Prognostic Values of the Eight Subunits of the Chaperonin TRiC in Breast Cancer

**DOI:** 10.3389/fgene.2021.637887

**Published:** 2021-03-17

**Authors:** Wen-Xiu Xu, Wei Song, Meng-Ping Jiang, Su-Jin Yang, Jian Zhang, Dan-Dan Wang, Jin-Hai Tang

**Affiliations:** Department of General Surgery, The First Affiliated Hospital of Nanjing Medical University, Nanjing, China

**Keywords:** bioinformatic analysis, breast cancer, gene expression, eight subunits of the chaperonin TRiC, prognosis

## Abstract

**Background:**

Chaperonin-containing TCP-1 (TRiC or CCT) was demonstrated to be involved in oncogenesis of cancers carcinogenesis and development of various malignancies. Increasing experimental evidence indicated that dysregulation of TRiC was implicated in the tumor progression of breast cancer (BCa). However, few definitive studies have addressed the diverse expression patterns and prognostic values of eight TRiC subunits. Thus, we aimed to investigate the clinical significance of TRiC subunit expression and prognostic values for their possible implications in diagnosis and treatment of BCa.

**Methods:**

Based on updated public resources and comprehensive bioinformatics analysis, we used some online databases (e.g., UALCAN, GEPIA, cBioPortal, TIMER, BC-GenExMiner, metascape, and GeneMANIA) to comprehensively explore the expression levels and the prognostic effects of eight TRiC subunits in patients with BCa.

**Results:**

The transcriptional levels of most subunits of the Chaperonin TRiC (CCT2, CCT3, CCT4, CCT5, CCT6A, and CCT7) were significantly elevated compared with normal breast tissues, whereas TCP1, CCT4, and CCT6B were lower in BCa tissues than in normal tissues. Besides, copy-number alterations (CNA) of eight TRiC subunits positively regulated their mRNA expressions. Furthermore, high mRNA expression of TCP1/CCT2/CCT4/CCT5/CCT6A/CCT7/CCT8 was significantly associated with poor overall survival (OS) in BCa patients. The eight subunits of the chaperonin TRiC was related to tumor purity and immune infiltration levels of BCa. Co-expression analysis showed CCT6B was negatively associated with other subunits of TRiC and other subunits of TRiC were positively correlated with each other. Additionally, TRiC and their interactive proteins were correlated with positive regulation of biological process, localization, and biological regulation.

**Conclusion:**

This study systematically illustrated the expression profiles and distinct prognostic values of chaperonin TRiC in BCa, providing insights for further investigation of subunits of the chaperonin TRiC as novel therapeutic targets and potential prognostic biomarkers in BCa.

## Introduction

Breast cancer (BCa) ranks first in terms of morbidity and is the second leading cause of mortality among all female’s cancers globally, and 276,480 new cancer cases and 42,170 cancer deaths are expected in United States in 2020 according to the estimation by the American Cancer Society (Siegel et al., 2020). Surgery and chemotherapy are curative treatments in early-stage BCa patients. However, a considerable proportion of BCa patients are not diagnosed or treated until they reach an advanced stage, resulting in a poor prognosis (Zheng et al., 2018). In such circumstances, identification of novel biomarkers, and treatment targets are needed to better delineate patient outcomes and individualize patient management in BCa.

Chaperonins are molecules that mediates nascent polypeptide chains folding and include two groups, group I and group II. Heat shock protein 60 (HSP60) or bacteria GroEL belongs to group I, and CCT [chaperonin-containing t-complex polypeptide 1 (TCP-1) or TRiC] belongs to group II. The eukaryotic cytoplasmic CCT complexes are assembled into two symmetry rings, each of which consists of eight paralogous but distinct subunits, denoted TCP1, CCT2, CCT3, CCT4, CCT5, CCT6A, CCT6B, CCT7, and CCT8 (or CCTα, CCTβ, CCTγ, CCTδ, CCTε, CCTζ-1, CCTζ-2, CCTη, and CCTθ) (Shimon et al., 2008; Amit et al., 2010). TRiC, also known as CCT, is essential for cell viability and has been shown to assist the folding of cytoskeletal proteins (tubulins and actins) and other proteins related to carcinogenesis, such as p53, STAT3 (Signal transducer and activator of transcription 3) in an ATP-dependent fashion (Amit et al., 2010; Trinidad et al., 2013; Kasembeli et al., 2014; Gestaut et al., 2019). Under such circumstances, elevated expression of TRiC subunits may lead to upregulation of these oncogenic proteins, concomitantly resulting in carcinogenesis. Except for CCT6B, other TRiC subunits, TCP1, CCT2, CCT3, CCT4, CCT5, CCT6A, CCT7, and CCT8 exhibit approximately 30% identity. Intriguingly, CCT6B is the most special one among different TRiC subunits, and is expressed only in testis, which may play a specific role in helping the biosynthesis of particular testicular proteins (Kubota et al., 1997).

In the current research, the subunits of CCT have been shown to be critical for the development and progression of BCa (Guest et al., 2015). Intriguingly, we investigated and found aberrant expressions of the TRiC subunits in BCa, including significantly elevated expressions of TCP1, CCT2, CCT3, CCT4, CCT5, CCT6A, CCT7, and CCT8 as well as decreased expression of CCT6B, which were determinants of growth and overall survival (OS) in BCa. Our results demonstrated a role for the TRiC subunits, suggesting that the role of the entire complex potentially could be explored as a functional macrocosm in BCa.

## Materials and Methods

### UALCAN Database Analysis

UALCAN web-portal^1^ is an interactive and effective platform based on level 3 RNA-seq and clinical information from The Cancer Genome Atlas (TCGA) project (Chandrashekar et al., 2017). It can be utilized to analyze relative transcriptional levels of target genes between cancerous and paired normal tissues. In the current study, we explored the relative expression of eight subunits of the chaperonin TRiC between BCa and paracancerous tissues and compared the expression differences among different molecular subtypes based on UALCAN database. All the BCa cases publicly available on UALCAN were included in our research.

### cBioPortal Database Analysis

cBioPortal^2^ is a user-friendly, comprehensive website resource and provides visualization, analysis, and download of large-scale cancer genomics datasets (Wu et al., 2019). In our study, we analyzed the genetic alterations of TRiC, which contained genomic profiles counted on mutations and putative copy-number alterations (CNA) from GISTIC. Breast Invasive Carcinoma (TCGA, Cell 2015) was selected for further analysis of TRiC, and tumor samples included in our research contained total 817 BCa samples. OncoPrint was constructed in cBioPortal to directly reflect all types of changes in eight subunits of the chaperonin TRiC gene amplification, deep deletion, mRNA upregulation, and mRNA downregulation in patients with BCa. Furthermore, we downloaded the data of putative CNAs and mRNA expression *z*-scores to evaluate the association between various CNAs and transcriptional levels of chaperonin TRiC.

### GEPIA Database Analysis

The Gene Expression Profiling Interactive Analysis (GEPIA) database^3^, a newly developed web server for analyzing the of RNA sequencing data based on 9,736 tumors and 8,587 normal samples from TCGA and GTEx projects. It provides key interactive and customizable functions including differential expression analysis, correlation analysis, profiling plotting, similar gene detection, patient survival analysis, and dimensionality reduction analysis (Tang et al., 2017). The Survival Analysis module on GEPIA was applied to estimate the correlation between TRiC expression and survival information of BCa patients. The prognostic values of chaperonin TRiC (TCP1, CCT2, CCT3, CCT4, CCT5, CCT6A, CCT6B, CCT7, and CCT8) at mRNA level were analyzed using all BCa samples available in GEPIA. The patients’ cohorts were split at the median expression of each subunit of the chaperonin TRiC mRNA level. Hazard ratio (HR) and log-rank *P*-value were calculated and displayed online. Meanwhile, the relationship between the expression level of chaperonin TRiC and gene markers of tumor-infiltrating immune cells (TIICs) was also explored in GEPIA. These markers were used to characterize immune cells, including CD8+ T cell, T cell (general), B cell, Tfh, M1, M2, and TAM (tumor-associated macrophages) in BCa.

### TIMER Database Analysis

Tumor Immune Estimation Resource database (TIMER^4^) includes more than 10,000 samples across 32 cancer types from TCGA, which is an easy-to-operate online tool established for systematically analyzing the abundance of immune infiltration (Li et al., 2016, 2017). In our research, we mainly explored the correlation of TRiC expression with the abundance of all six immune infiltration fluids in BCa, including B cells, CD4+ T cells, CD8+ T cells, neutrophils, macrophages, and dendritic cells (DCs).

### BC-GenExMiner Database Analysis

Breast cancer Gene-Expression Miner (bc-GenExMiner v4.5)^5^, a mining tool of annotated genomics data, provides biologists with prognostic analysis and may be conducted on cohorts split by estrogen receptor (ER), nodal (N), or molecular subtype status (Jézéquel et al., 2012). This online tool also allowed Pearson correlation analysis of eight subunits of the chaperonin TRiC in BCa.

### Protein–Protein Interaction and Functional Enrichment Analysis

GeneMANIA^6^ is a user-friendly online tool that can be adopted to derive hypotheses based on gene functions (Warde-Farley et al., 2010). It generated a list of genes with similar functions and constructed an interactive functional-association network to elucidate relationships between genes and datasets. In our study, this database was applied to construct a PPI network for eight subunits of the chaperonin TRiC to evaluate their functions. Metascape^7^ provides a flexible web interface for systematic and comprehensive functional annotation and analysis to aid investigators identify the biological meaning behind an extensive list of genes (Zhou et al., 2019). In this study, we used it to perform enrichment analysis of eight subunits of the chaperonin TRiC and their identified co-expression genes.

### Statistical Analysis

The differential mRNA expression of TRiC in BCa tissues from the UALCAN database was analyzed by Student’s *t*-test and normalized as transcripts per million reads (TPM). Survival curves were generated and compared by log-rank test. The correlation analysis was evaluated in the GEPIA database using Spearman’s correlation analysis. Differences were considered statistically significant when P-values were and/or equal to 0.05.

## Results

### Transcriptional Levels of the Eight Subunits of TRiC in BCa

**FIGURE 1:**
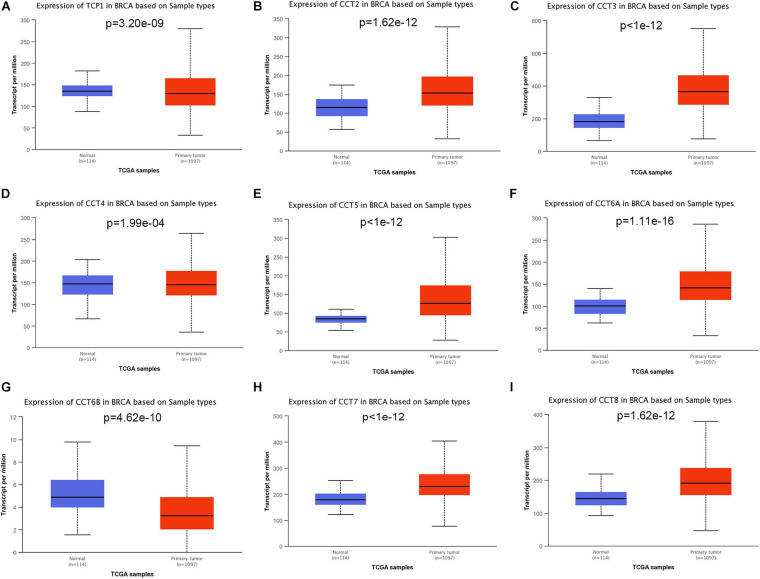
Boxplot showed relative expression of eight subunits of the chaperonin TRiC in BCa primary tumor and in corresponding normal tissues based on UACLAN database. **(A)** TCP1, **(B)** CCT2, **(C)** CCT3, **(D)** CCT4, **(E)** CCT5, **(F)** CCT6A, **(G)** CCT6B, **(H)** CCT7, and **(I)** CCT8.

**FIGURE 2:**
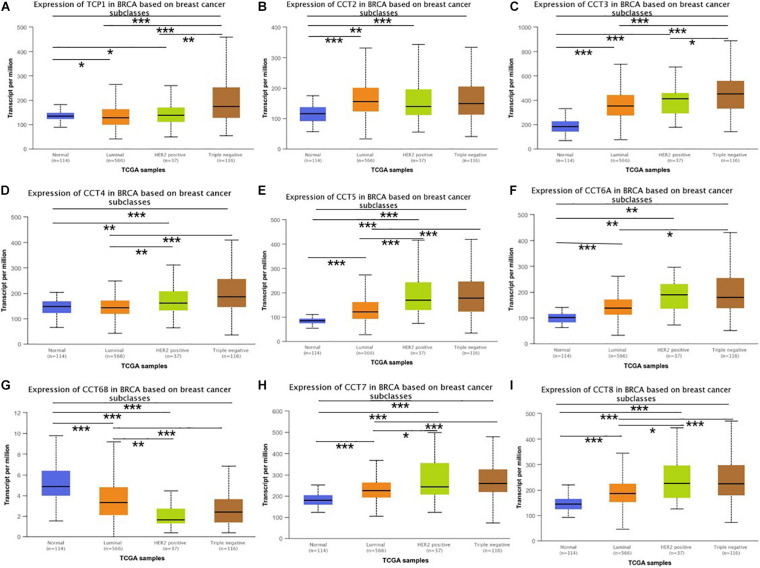
Boxplot showed relative expression of eight subunits of the chaperonin TRiC in BCa patients with different molecular subtypes and in normal individuals based on UACLAN database. **(A)** TCP1, **(B)** CCT2, **(C)** CCT3, **(D)** CCT4, **(E)** CCT5, **(F)** CCT6A, **(G)** CCT6B, **(H)** CCT7, and **(I)** CCT8. *p < 0.05; **p < 0.01; ***p < 0.001.

To explore the exact expression profiles of TRiC in BCa patients, we compared their differential transcriptional levels between BCa and normal samples by using UALCAN database. The findings revealed that the expression of five genes was higher in BCa samples than in normal control samples. As shown in Figure 1, the mRNA expression levels of CCT2 (Figure 1B, *P* < 0.001), CCT3 (Figure 1C, *P* < 0.001), CCT5 (Figure 1F, *P* < 0.001), CCT6A (Figure 1E, *P* < 0.001), CCT7 (Figure 1H, *P* < 0.001), and CCT8 (Figure 1I, *P* < 0.001) was significantly upregulated in BCa tissues compared with paracancerous tissues. Besides, the transcriptional level of TCP1 (Figure 1A, *P* < 0.001), CCT4 (Figure 1D, *P* < 0.001) and CCT6B (Figure 1G, *P* < 0.001) was significantly downregulated in BCa tissues compared with paracancerous tissues. Further, when sorting the patients by subgroups, all subunits of the chaperonin TRiC were still significantly up-regulated in different molecular subtypes compared with paracancerous samples, except for CCT6B, which was down-regulated BCa patients (Figure 2G). Additionally, the highest expression levels of TCP1, CCT3, CCT4, CCT5, and CCT7 were observed in triple-negative BCa tissues (Figures 2A,C–E,H). CCT6A and CCT8 was enriched in HER2-positive BCa tissues (Figures 2F,I), and CCT2 was enriched in luminal BCa tissues (Figure 2B).

### Genetic Alterations of the Eight Subunits of TRiC in BCa Samples

**FIGURE 3:**
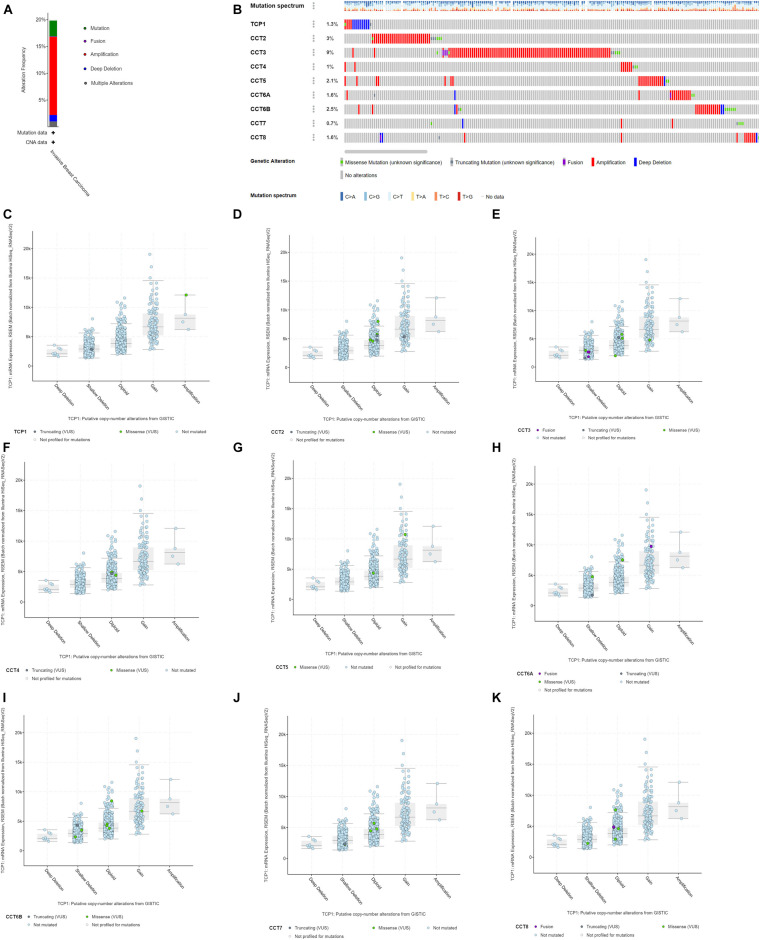
Genetic mutation analysis of eight subunits of the chaperonin TRiC in BCa (cBioPortal). **(A)** Types of mutations and their proportions contained in BCa. **(B)** OncoPrint visual summary of genetic alteration on a query of eight subunits of the chaperonin TRiC in BCa. TCP1, CCT2, CCT3, CCT4, CCT5, CCT6A, CCT6B, CCT7, and CCT8 mutation rates were 1.3, 3, 9, 1, 2.1, 1.6, 2.5, 0.7, and 1.6%, respectively. **(C–K)** Copy gain (gain and amplification) of TRiC was linked with significant upregulated TRiC mRNA levels compared with the copy-loss (deep deletion and shallow deletion) and copy-neutral (diploid) patients **(C)** TCP1, **(D)** CCT2, **(E)** CCT3, **(F)** CCT4, **(G)** CCT5, **(H)** CCT6A, **(I)** CCT6B, **(J)** CCT7, and **(K)** CCT8.

Next, the genetic alterations of TRiC in BCa patients were explored based on TCGA database and cBioPortal online tool. The frequencies of mutations of the eight subunits of TRiC genes in breast invasive carcinoma were assessed using cBioPortal database. DNA copy number amplifications, mutations, and deep deletion were the main genetic mutations of BCa (Figure 3A). As shown in Figure 2B, the percentages of genetic variations in the eight subunits of TRiC among BCa patients varied from 0.7 to 9% for individual genes (TCP1, 1.3%; CCT2, 3%; CCT3, 9%; CCT4, 1%; CCT5, 2.1%, CCT6A, 1.6%; CC6B, 2.5%, CCT7, 0.7%, and CCT8, 1.6%, respectively). CCT2, CCT3, and CCT6B were ranked as the top three of the eight members. DNA CNA are most common genetic alterations which are involved in carcinogenesis through modulating cancer-related gene expression (Thomas et al., 2015; Choi et al., 2017). The eight subunits of TRiC were dysregulated in BCa tissues, we further hypothesized that DNA CNA might modulate their transcriptional levels. As shown in Figure 3, low amplification rate of the eight subunits of TRiC was observed in BCa patients. However, although copy gain (gain and amplification) of eight subunits of TRiC was not frequent, it was still linked with significant upregulated eight subunits of TRiC mRNA levels compared with the copy-loss (deep deletion and shallow deletion) and copy-neutral (diploid) patients (Figures 3C–K). To sum up, these results indicated that eight subunits of TRiC mRNA expressions were modulated by their DNA CNA.

### Prognostic Values of TRiC mRNA Expression in All BCa Samples

**FIGURE 4:**
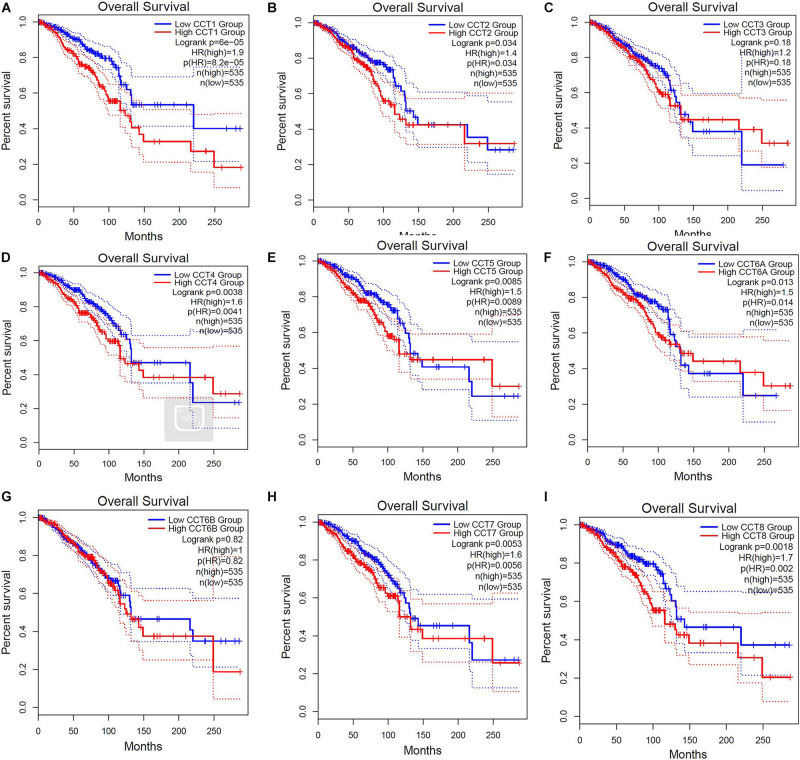
Elevated expressions of eight subunits of the chaperonin TRiC were associated with poor clinical outcomes (OS). Prognostic value of mRNA level of TRiC in BCa patients **(A)** TCP1, **(B)** CCT2, **(C)** CCT3, **(D)** CCT4, **(E)** CCT5, **(F)** CCT6A, **(G)** CCT6B, **(H)** CCT7, and **(I)** CCT8.

Further, we employed GEPIA database to analyze the associations between TRiC mRNA expression and prognosis of BCa patients. As shown in Figure 4, high mRNA expression of TCP1 (HR = 1.9, *P* < 0.001), CCT2 (HR = 1.4, *P* = 0.034), CCT4 (HR = 1.6, *P* = 0.0041), CCT5 (HR = 1.5, *P* = 0.0089), CCT6A (HR = 1.5, *P* = 0.014), CCT7 (HR = 1.6, *P* = 0.0056), and CCT8 (HR = 1.7, *P* = 0.002) were significantly associated with poor OS of BCa patients. However, other subunits of TRiC mRNA expression showed a null correlation with prognosis of BCa patients. Overall, our findings above implied that mRNA expressions of TCP1, CCT2, CCT4, CCT5, CCT6A, CCT7, and CCT8 were remarkably correlated with BCa patients’ OS, which might be identified as promising biomarkers to predict the survival of BCa patients.

### Relationships Between TRiC Expression and Immune Infiltration

**FIGURE 5:**
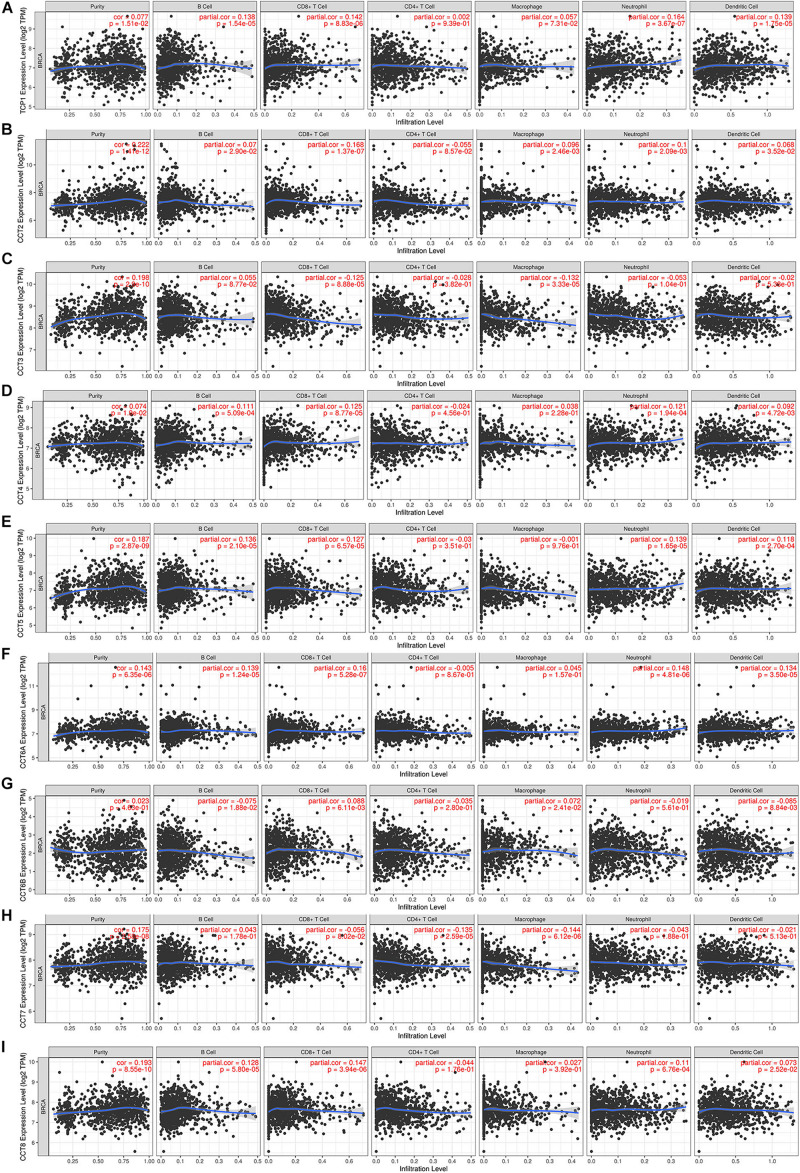
Correlation of eight subunits of the chaperonin TRiC with immune infiltration level in BCa. **(A)** TCP1 expression had significant positive correlations with infiltrating levels of CD4+ T cells. **(B)** CCT2 expression had significant correlations with infiltrating levels of CD4+ T cells, macrophages, neutrophils, and dendritic cells in GC. **(C)** CCT3 expression was significantly related to infiltrating levels of CD4+ T cells and macrophages in GC but no significant correlation with infiltrating level of B cells. **(D)** CCT4 expression had significant positive correlations with infiltrating levels of CD4+ T cells and macrophages in GC. **(E)** CCT5, **(F)** CCT6A, **(G)** CCT6B, **(H)** CCT7, and **(I)** CCT8.

Immune cells in the TME can affect patient survival, and the prognosis of patients with BCa is closely related to the infiltration of immune cells (Shen et al., 2020). The above findings indicated a prognostic role of TRiC in BCa. Hence, it would be meaningful to explore the association between immune infiltration and TRiC expression. We determined whether TRiC expression was correlated with the immune infiltration level in different cancers by calculating the coefficient of TRiC expression and immune infiltration level in BCa in TIMER. As distinctly shown in Figure 5, the results revealed that TRiC expression had positive correlations with tumor purity and CD8+ T cells except CCT6B. CCT2 was positively associated with macrophage, while CCT3 was negatively associated with macrophage. Furthermore, TCP1, CCT2, CCT4, CCT5, CCT6A, and CCT8 were positively associated with neutrophil, B cell and DC, while CCT6B was negatively associated with DC. These results showed the eight subunits of the chaperonin TRiC was related to tumor purity and immune infiltration levels of BCa by TIMER analysis.

### Relationships Between TriC Expression and Immune Markers

Accumulating evidence showed that the interaction between cancer cells and the tumor microenvironment, specifically the immune microenvironment, was also believed to be a vital factor and involved in the tumor progression and therapy (Hirsch et al., 2017). So as to further explore the potential relationships between TRiC and infiltrating immune cells, we examined the correlations between eight subunits of TRiC and several immune cell markers in GEPIA. Immune genes were selected from ImmPort database^8^ (Zalocusky et al., 2018). These markers were used to characterize immune cells, including CD8A, CD8B, CD3D, CD3E, CD2, CD19, CD79A, BCL6, NOS2, ROS, ARG1, MRC1, HLA-G, CD80, and CD86 in BCa (Table 1). Most subunits of TRiC were positively correlated with CD8+ T cell markers (CD8A and CD8B) in breast normal tissues. Hence, these results confirmed our speculation that TRiC expression in BCa was correlated with gene markers of immune cells in different manners, which can help explain the differences in patient survival.

**TABLE 1:** Correlation analysis between TRiC and genes markers of immune cells in GEPIA.

**Description**			**CD8+ T cell**		**T cell (general)**			**B cell**		**Tfh**	**M1**		**M2**		**TAM**		
**Gene markers**			**CD8A**	**CD8B**	**CD3D**	**CD3E**	**CD2**	**CD19**	**CD79A**	**BCL6**	**NOS2**	**ROS**	**ARG1**	**MRC1**	**HLA-G**	**CD80**	**CD86**
**TCP1**	Tumor	*R*	–0.039	0.014	–0.092	–0.072	–0.017	–0.088	–0.1	–0.078	0.024	0.12	–0.031	0.019	–0.0011	0.17	0.11
		*P*	0.19	0.64	*	0.018	0.59	*	**	0.01	0.43	***	0.3	0.52	0.97	***	***
	Normal	*R*	0.43	0.38	0.17	0.19	0.27	–0.016	0.038	–0.057	0.15	–0.14	0.3	–0.13	–0.035	0.18	0.022
		*P*	***	***	0.066	0.046	*	0.87	0.69	0.55	0.11	0.14	*	0.16	0.72	0.056	0.82
**CCT2**	Tumor	*R*	–0.058	–0.05	–0.1	–0.09	–0.069	–0.082	–0.1	–0.032	–0.025	0.015	–0.019	–0.019	–0.028	0.011	–0.031
		*P*	0.056	0.1	**	*	0.022	*	**	0.29	0.4	0.63	0.53	0.54	0.35	0.72	0.31
	Normal	*R*	0.42	0.37	0.21	0.19	0.22	0.022	0.09	–0.17	0.061	–0.12	0.27	–0.23	0.0058	0.22	–0.0071
		*P*	***	***	0.023	0.041	0.02	0.81	0.34	0.067	0.52	0.21	*	0.016	0.95	0.019	0.94
**CCT3**	Tumor	*R*	–0.11	0.056	–0.15	–0.15	–0.11	–0.15	–0.19	–0.074	0.018	–0.0019	0.0033	–0.06	0.043	0.081	–0.04
		*P*	**	0.064	***	***	**	***	***	0.015	0.56	0.95	0.91	0.048	0.16	*	0.18
	Normal	*R*	0.45	0.53	0.32	0.3	0.3	0.09	0.18	–0.29	0.072	–0.13	0.27	–0.46	0.15	0.19	–0.17
		*P*	***	***	**	**	*	0.35	0.061	*	0.45	0.19	*	***	0.11	0.049	0.07
**CCT4**	Tumor	*R*	–0.041	0.056	–0.082	–0.084	–0.028	–0.1	–0.12	–0.15	0.054	0.085	–0.037	0.026	0.02	0.15	0.067
		*P*	0.17	0.064	*	*	0.35	**	***	***	0.075	*	0.22	0.39	0.5	***	0.027
	Normal	*R*	0.5	0.53	0.26	0.31	0.32	–0.0042	0.095	–0.31	0.051	–0.13	0.3	–0.45	–0.0014	0.067	0.19
		*P*	***	***	*	*	**	0.96	0.32	***	0.59	0.17	*	***	0.89	0.48	0.043
**CCT5**	Tumor	*R*	–0.031	0.036	–0.094	–0.079	–0.031	–0.088	–0.11	–0.041	0.053	0.062	–0.014	–0.014	0.15	0.19	0.095
		*P*	0.31	0.23	*	*	0.31	*	**	0.18	0.079	0.041	0.64	0.65	***	***	*
	Normal	*R*	0.27	0.22	0.17	0.17	0.18	0.09	0.11	–0.012	0.0042	0.05	0.12	0.052	0.14	0.29	0.19
		*P*	*	0.019	0.066	0.081	0.058	0.35	0.25	0.9	0.96	0.6	0.19	0.58	0.15	*	0.043
**CCT6A**	Tumor	*R*	–0.033	–0.0098	–0.032	–0.031	–0.012	–0.039	–0.044	–0.063	0.0036	0.0076	–0.0088	–0.012	–0.0018	0.038	0.036
		*P*	0.28	0.75	0.29	0.3	0.7	0.2	0.15	0.037	0.9	0.8	0.77	0.7	0.95	0.21	0.24
	Normal	*R*	0.54	0.49	0.37	0.37	0.4	0.11	0.16	–0.36	–0.052	0.05	0.23	–0.28	0.092	0.27	0.096
		*P*	***	***	***	***	***	0.24	0.086	***	0.58	0.6	0.014	*	0.33	*	0.31
**CCT6B**	Tumor	*R*	–0.096	–0.099	–0.14	–0.12	–0.12	0.0011	–0.13	0.12	–0.011	–0.027	–0.01	0.0068	–0.03	–0.079	–0.068
		*P*	*	*	***	***	***	–0.099	***	***	0.72	0.38	0.73	0.82	0.33	*	0.025
	Normal	*R*	0.39	0.4	0.17	0.18	0.18	0.021	0.31	–0.22	0.24	–0.18	0.26	–0.41	0.01	0.082	–0.15
		*P*	***	***	0.069	0.054	0.054	0.83	0.097	0.019	0.012	0.064	*	***	0.92	0.39	0.11
**CCT7**	Tumor	*R*	–0.11	–0.026	–0.14	–0.15	–0.12	–0.14	–0.17	–0.2	0.063	0.052	–0.0082	–0.045	0.012	0.044	–0.083
		*P*	**	0.39	***	***	***	***	***	***	0.038	0.09	0.79	0.14	0.7	0.15	*
	Normal	*R*	0.28	0.39	0.18	0.16	0.15	–0.037	0.03	–0.4	0.012	–0.078	0.15	–0.35	0.11	0.03	–0.13
		*P*	*	***	0.053	0.091	0.12	0.7	0.75	***	0.9	0.41	0.11	***	0.26	0.75	0.16
**CCT8**	Tumor	*R*	–0.084	–0.055	–0.13	–0.11	–0.062	–0.12	–0.15	–0.081	0.012	0.069	0.0017	0.0034	–0.0033	0.11	0.059
		*P*	*	0.069	***	**	0.042	***	***	*	0.68	0.024	0.96	0.91	0.91	***	0.054
	Normal	*R*	0.54	2.1*e*−07	0.34	0.35	0.4	0.13	0.18	–0.17	0.056	–0.15	0.36	–0.35	0.076	0.22	–0.059
		*P*	***	0.47	**	**	***	0.17	0.055	0.07	0.56	0.11	***	***	0.43	0.022	0.54

Tfh, follicular helper T cell; TAM, tumor-correlated macrophage; *R*, *R*-value of Spearman’s correlation; *P*, *P*-value of Spearman’s correlation. *P < 0.01; **P < 0.001; ***P < 0.0001.

### Enrichment Analysis of Protein–Protein Interaction of TriC

**FIGURE 6:**
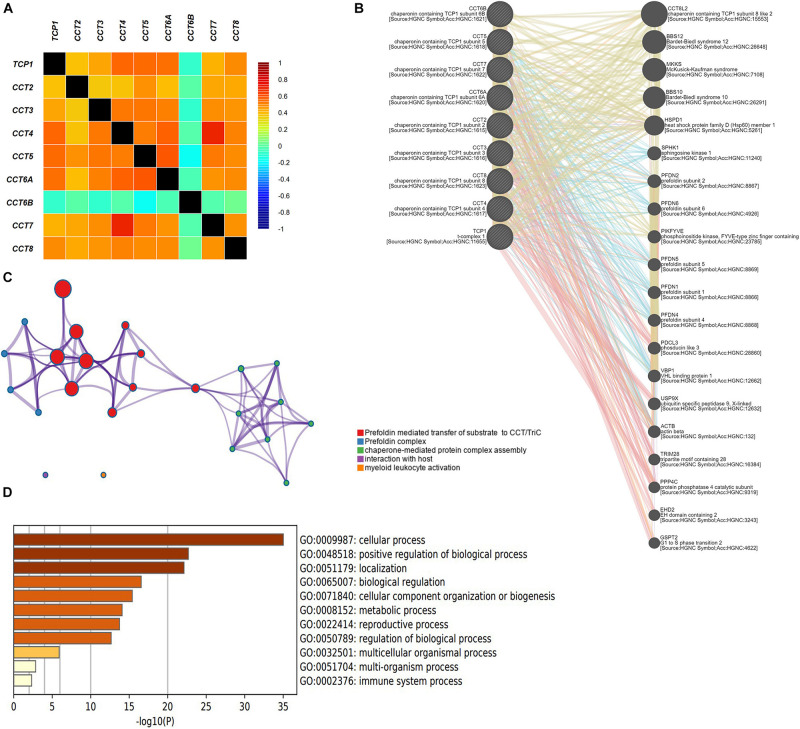
Enrichment analysis **(A)** Pearson correlation analysis of eight subunits of the chaperonin TRiC. **(B)** Protein–protein interaction network among eight subunits of the chaperonin TRiC in the GeneMANIA dataset. **(C)** Interactive network of the top five enriched terms colored by cluster ID. Each color represents one enrichment pathway. **(D)** Heatmap of enriched terms regarding Gene Ontology biological processes across eight subunits of the chaperonin TRiC and its co-expressed genes constructed by Metascape.

As shown in Figure 6A, it revealed a significant negative correlation between CCT6B and other subunits of TRiC. Furthermore, other subunits of TRiC were correlated to a significant degree. A network of eight subunits of TRiC and 20 proteins that significantly interacted with TRiC was constructed using GeneMANIA. The results revealed that BBS12, MKKS, BBS10, HSPD1, SPHK1, PFDN2, PFDN6, PIKFYVE, PFDN5, PFDN1, PFDN4, PDCL3, VBP1, USP9X, ACTB, TRIM28, PPP4C, EHD2, and GSPT2 were closely associated with eight subunits of TRiC (Figure 6B). Next, a PPI enrichment analysis, was then used to explore the relationships among these genes in BCa. The functions of these genes were next explored through GO analyses (Figures 6C,D). GO analyses allow assessment of the biological process, molecular function, and cellular component annotations of genes of interest. These 29 genes were primarily enriched for regulation of cellular process, positive regulation of biological process, localization, biological regulation, cellular component organization or biogenesis, metabolic process, reproductive process, and regulation of biological process.

## Discussion

Chaperonin-containing TCP-1, a multi-subunit complex and encoded by eight distinct genes, folds various proteins that essential for cancer development, and is expressed in diverse cancers and can serve as a viable therapeutic target (Showalter et al., 2020). Previous studies reported that the expression levels of different CCT subunits were upregulated in various cancers, such as CCT2 in prostate, breast, and lung cancers (Guest et al., 2015; Bassiouni et al., 2016; Carr et al., 2017), CCT3 in hepatocellular carcinoma (HCC) (Qian et al., 2016), and CCT8 in HCC and glioblastoma (Huang et al., 2014; Qiu et al., 2015). CCT2 played a pivotal role in clinical tubulin-binding agent-resistant or CCT2-overexpressing cancers, and targeting the β-tubulin/CCT2 complex might provide these cancers with a novel chemotherapeutic strategy (Lin et al., 2009). These pathways proceeded through activating mitogen-activated protein kinases (MAPKs) at the onset of β-tubulin/CCT2 complex disruption (Liu et al., 2017). Besides, targeting the complex also induced apoptosis and inhibited migration and invasion of metastatic human lung adenocarcinoma (Liu et al., 2020). CCT2 was a vital determinant of survival in CRC (colorectal cancer) patients and could regulate the folding of Gli-1, a Hedgehog signaling factor in relation to hypoxia (Park et al., 2020). CCT3 functioned as a trigger of YAP and TFCP2 to affect tumorigenesis and served as a potential biomarker in liver cancer (Liu et al., 2019). Furthermore, CCT3 was closely related to the proliferation and migration of BCa and papillary thyroid carcinoma (PTC) (Shi et al., 2018; Xu et al., 2020). The correlation between the aberrant overexpression of CCT3 and the poor prognosis of HCC patients has been shown, as has the depletion of CCT3 sensitized HCC cells to chemotherapy (Zhang et al., 2016). MALAT-1 enhanced cell motility of lung adenocarcinoma by downregulating CCT4 (Tano et al., 2010). CCT5 was a tumor associated antigen of non-small cell lung cancer (NSCLC) (Gao et al., 2017). CCT6A sustained the oncogenic arm of TGF-β signaling and functioned as a potent promoter of TGF-β-induced metastasis of NSCLC cells, blocking SMAD2-SMAD4 interaction (Ying et al., 2017). Increased CCT8 expression was associated with poor prognosis and cisplatin resistance by regulating α-actin and β-tubulin in ESCC (Yang et al., 2018). Compared with the controls, the glioma cells expressing CCT8-siRNA exhibited a significantly decreased proliferation and invasion capacity (Qiu et al., 2015). Another study defined CCT8 as an oncogene and demonstrated its function of participating in HCC cell proliferation by facilitating S-phase entry (Huang et al., 2014). Overexpression of CCT8 could promote the proliferation, accelerate the G1/S transition and reverse cell adhesion-mediated drug resistance (CAM-DR) phenotype in B-cell non-Hodgkin’s lymphoma (Yin et al., 2016). Tumor infiltrates consist of a heterogeneous population of immune cells frequently dominated by T cells but also containing B cells, macrophages, NK cells, DCs, and neutrophils, which play vital roles in anti-tumor immunity (Garaud et al., 2019). Tumor-infiltrating lymphocytes (TILs) and tumor-infiltrating B-cells (TIL-B) are crucial determinants of favorable outcomes in patients with BCa (Garaud et al., 2019; Byrne et al., 2020). Immunotherapy using immune cells-based vaccination is a promising approach to eliminate tumor cells (Sabado et al., 2017).

Here, we explored the role and prognostic value of the full chaperonin TRiC in BCa by studying the altered expression of each of its subunits in the context of this disease. Furthermore, we also found that the chaperonin TRiC subunits can act as mediators only in certain states, such as when associated with ATP. Chaperonin TRiC was thus a viable target for therapeutic intervention in cancer due to its function as a critical protein-folding complex. Overall, our research preliminarily but systematically characterized the expression profiles of eight subunits of TRiC in BCa and revealed that the detection of the TRiC expression status of BCa patients might be promising and valuable biomarkers for early diagnosis, immunotherapy, and prognostic assessment.

## Conclusion

In summary, our results indicated that subunits of TRiC displayed varying degrees of abnormal expressions, and CCT2, CCT3, CCT5, CCT6A, CCT7, and CCT8 were significantly upregulated in BCa patients and their upregulation was positively correlated with BCa tumor stage. Based on the above findings, it was expected that TRiC could act as potential prognostic biomarkers and therapeutic targets for BCa. Our research contributed to a better understanding of the pathogenesis of BCa and might assist in the development of more effective targeted drugs for BCa. However, further relevant experimental studies were needed to validate our findings and to promote clinical application of TRiC as prognostic or therapeutic targets in BCa, owing to limited sample sizes and differences found among databases.

## Data Availability Statement

The original contributions presented in the study are included in the article/supplementary material, further inquiries can be directed to the corresponding author/s.

## Author Contributions

W-XX and WS conceived and designed this study. W-XX, WS, M-PJ, and S-JY acquired and downloaded the data. W-XX, WS, D-DW, and JZ analyzed the data. W-XX, WS, D-DW, JZ, and J-HT helped discuss the results. W-XX drafted the manuscript. All authors revised and reviewed this work, and gave their final approval of this manuscript.

## Conflict of Interest

The authors declare that the research was conducted in the absence of any commercial or financial relationships that could be construed as a potential conflict of interest.

## Footnotes

1 http://ualcan.path.uab.edu/

2 www.cbioportal.org/

3 http://gepia.cancer-pku.cn/

4 https://cistrome.shinyapps.io/timer/

5 http://bcgenex.centregauducheau.fr

6 http://www.genemania.org

7 http://metascape.org

8 https://immport.niaid.nih.gov
